# Supplementary material for: Serum-deprived differentiated neuroblastoma F-11 cells express functional dorsal root ganglion neuron properties
Source: PeerJ. 2019 Oct 30;7:e7951. doi: 10.7717/peerj.7951 (PMC6825413; doi:10.7717/peerj.7951)
Supplement: Supplemental Information 3 — TTX-S, tetrodotoxin-sensitive Na currents; TTX-R, TTX-resistant Na currents; N.A., not applicable. Data are expressed as mean ± SEM. Numbers in parentheses indicate number of cells or percentage of cells. Numbers in brackets indicate the references: [1] Sculptoreanu & de Groat (2007); [2] Yu et al. (2014); [3] Cummins & Waxman (1997); [4] Hall, Sima & Wiley (1995); [5] Carbone & Lux (1987); [6] Moraes, Kushmerick & Naves (2014); [7] Lei et al. (2014); [8] Genzen, Van Cleve & McGehee (2001); [9] Chiesa et al. (1997); [10] Kusano & Gainer (1993); [11] Ambrosino et al. (2013); [12] Raymon et al. (1999); [13] Chen et al. (2007). [file peerj-07-7951-s003.doc]

|  | **10% serum**  **F-11 cells** | **1% serum**  **F-11 cells** | **Isolated rat DRG neurons** | **Differentiated**  **F-11 cells** | **Differentiated human sensory cell line** [12] | **Sensory model cell line (differentiated rat 50B11)** [13] |
| --- | --- | --- | --- | --- | --- | --- |
| ***Capacitance (pF)*** | 31±6 (19) | 43±5 (37) | 20-90 [1], 22 [2] | 61-64 [9] | N.A. | N.A. |
| ***Vrest (mV)*** | -17.1±3.8 (19) | -50.5±1.9 (40) | -53±1 (30) [1] | -28.4±1.9 (36) [10] | N.A. | -58.3±2.7 (6) |
| ***INa (nA)***  ***TTX-S***    ***TTX-R*** | 1.9±0.9 (19)  0 | 4.5±0.6 (37)  0 | 28.7±4.8 (11)  22.1±3.0 (11) [3] | 0.93±0.68 [10] | 0.91 ± 0.16 (9)  0 | N.A. |
| ***IK (pA)*** | 1436±426 (19) | 6422±561 (37) | N.A. | Yes [9] | N.A. | N.A. |
| ***Ierg (pA)*** | 535±129 (14) | 2163±723 (8) | N.A. | Yes [9] | N.A. | N.A. |
| ***ICa (pA)***  ***High threshold***  ***Low threshold*** | 107 ± 26 (15)  0 | 203 ± 44 (16)  0 | 2400-4300 [4]  300 [5] | 156±27 (22); 165±25 (11)  187±17 (32) [10] | 345 ± 49 (7)  0 | N.A. |
| ***Capsaicin*** | 0 | 41 ± 9 pA (13) | 50-5000 pA (58-67%) [1] | Increase in intracellular [Ca^2+^] (105/260) [11] | 423 ± 96 pA (11/11) | Rapid rise in intracellular [Ca^2+^] |
| ***Substance P*** | 0 | 24.5 ± 3.3 pA (4) | Increase (10/72) & decrease (10/72) [6] in excitability | N.A. | N.A. | N.A. |
| ***Acid pH***  ***pH5***    ***pH6*** | <20 pA (6)  <20 pA (5) | 931 ± 131 pA (32)  1021 ± 181 pA (22) | (96/110) [7] | N.A. | N.A. | N.A. |
| ***Acetylcholine*** | 43.6 **±** 12 pA (14/14) | 136 ± 35 pA (34/38) | 108-1262 pA [8] | N.A. | N.A. | N.A. |
| ***Glutamate*** | (1/13) | 48 ± 17 pA (17) | N.A. | N.A. | N.A. | N.A. |
| ***Induced AP firing***  ***(Hz)*** | Tonic: 10±3 (2/24)  Phasic: 2.1±0.4 (4/24) | Tonic: 7.7±0.5 (32/40)  Phasic: 1.6±0.1 (8/40) | Tonic: 17  Phasic: 2.5 [1]  Tonic: 14  Phasic: 3 [2] | Tonic: 7-8 (retinoic acid for 48h, [9]) | Multiple APs  (100%) | Yes (5/6) |
| ***Spontaneous***  ***AP firing (Hz)*** | 1.2±1 (2/11) | 0.8±0.2 (20/36) | N.A. | Yes [10] | N.A. | No |
